# Eye banking: an introduction

**Published:** 2009-12

**Authors:** Gullapalli N Rao, Usha Gopinathan

**Affiliations:** Chairman, LV Prasad Eye Institute (LVPEI), LV Prasad Marg, Banjara Hills, Hyderabad, 500 034 India. Email: gnrao@lvpei.org; Associate Executive Director, LVPEI and Vice President, Eye Bank Association of India. Email: usha@lvpei.org

**Figure:**
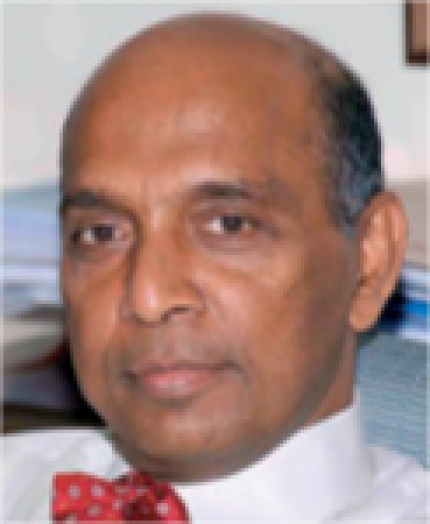


**Figure:**
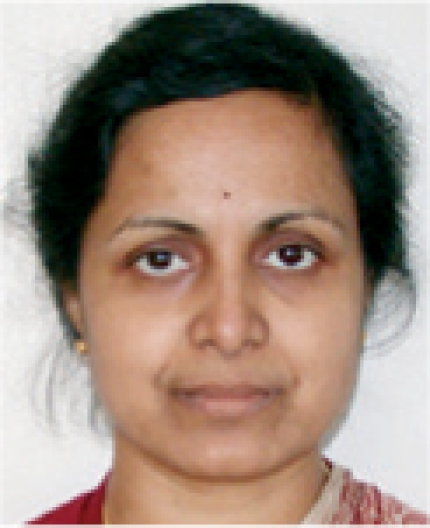


While prevention is the most desirable way to control corneal blindness, once a cornea has lost its transparency, a corneal transplant, or graft, is a patient's best chance to regain vision in the affected eye(s). However, the biggest limiting factor is the worldwide shortage of donated corneas.

In low- and middle-income countries, where the magnitude of corneal blindness is greatest, the availability of donated corneas is very low. This is due in large part to the lack of local eye banks. Efforts are under way to develop eye banks of optimal standards in many low- and middle-income countries, with countries like India and Philippines making notable progress. Myanmar, Ethiopia (see box right), and Kenya are examples where high quality eye banks have been established. However, this is still not enough to meet the need for corneas.

**The Eye Bank of Ethiopia**

**Figure:**
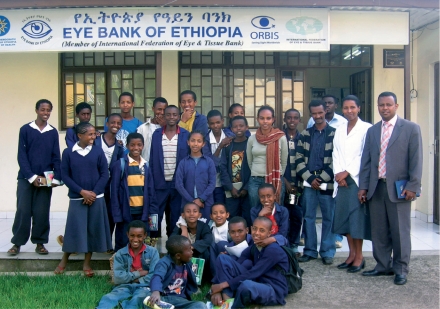
A group of children on a visit to the Eye Bank of Ethiopia

Eye Bank of Ethiopia

The Eye Bank of Ethiopia in Addis Ababa has been in existence since 2003. It is associated with Menelik II Referral Hospital, a tertiary referral centre, where most of the transplants are done. The eye bank also sends corneas to two university referral hospitals in northwestern and southern Ethiopia. Between 130 and 150 corneas are harvested (using in situ corneal excision) and used in 90–120 transplants every year. There are five corneal transplant surgeons in Ethiopia. Cornea donation is encouraged in a variety of ways, including media campaigns with well-known personalities such as the president of Ethiopia and athlete Haile Gabreselassi. So far, 6,000 Ethiopians, including Mr Gabreselassi, have pledged their corneas, and next-of-kin consent is being used increasingly. The eye bank is funded by ORBIS International Ethiopia and Addis Ababa City Government Health Bureau; it also raises funds locally. (Elmien Wolvaardt Ellison)

**Figure:**
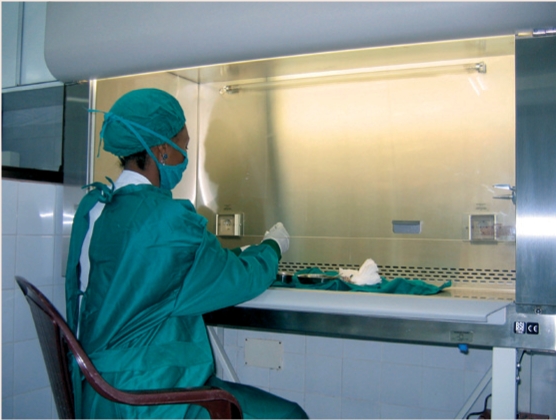
A technician at work in the Eye Eye Bank of Ethiopia

Eye Bank of Ethiopia

## What is an eye bank?

Eye banks are the institutions responsible for collecting (harvesting) and processing donor corneas, and for distributing them to trained corneal graft surgeons.^1^ Eye banks are regulated and part of the local health system; they may be attached to a hospital or housed in a separate building.

Cornea harvesting is the surgical removal from a deceased person of either the whole eye (enucleation) or the cornea (in situ corneal excision). This can be done by appropriately trained eye care personnel (eye bank technicians, ophthalmology residents, ophthalmologists, or general practitioners) in a variety of settings, including hospitals, homes, and funeral grounds.

### Before harvesting

Corneas can be harvested up to twelve hours after death, but ideally within six hours. The person who will harvest the cornea must first do the following:

- Obtain written consent from the senior next of kin of the deceased.
- Verify the death certificate and ensure there is a stated cause of death.
- Review the donor's medical and social history to ensure they have no contraindications to donation. (This is done by studying medical records, interviewing the physician under whose care the donor was, and interviewing close family members. Each eye bank must have a list of such contraindications, which are available from other well-established eye banks.)
- Obtain information about any blood loss occurred prior to and at time of death, and whether the donor received infusion/transfusion of crystalloids, colloids, and blood; these are used to calculate plasma dilution.

### During harvesting

Aseptic methods must be adhered to, including maintaining a sterile field while performing enucleation or in-situ corneal excision.^2^ Standard protocols include:

- pen torch examination of the eyes for foreign objects and other defects
- preparing the face and eyes of the donor using povidone iodine
- employing aseptic techniques for in situ corneal excision or enucleation
- immediate preservation of the excised eye or cornea in an appropriate cornea preservation medium
- drawing blood to screen the donor for infectious diseases. Each eye bank must decide the most appropriate serological tests needed but at a minimum they must test for HIV, hepatits B, and syphilis.

### Storing donated corneas

Whole eyes can be stored in a moist chamber at two to eight degrees Celsius. This is the simplest and least expensive way to store whole eyes, but the eyes have to be used within 48 hours. Such a storage method may be suitable for some eye banks with limited resources.

Excised corneas can be stored in intermediate-term preservation media, such as McCary Kaufman medium (MK medium) or Optisol, both maintained at four degrees Celsius. Corneas can be stored for 96 hours in the MK medium and ten days in Optisol.

With the availability of MK medium and Optisol, eye banks should ideally switch over from enucleation to in situ corneal excision procedures. This will enable better viability of donated corneas during storage. With increased resistance to the antibiotics used in preservation media, inclusion of alternative antibiotics must be considered.^3^

After corneas reach the eye bank, they are examined using a slit lamp to check for corneal and stromal pathology. The endothelial cell density is also examined by specular microscope; this is necessary as donor corneas with a low number of endothelial cells are likely to fail soon after surgery. The processing of whole eyes must be done within a laminar flow hood maintained in sterile conditions.

The suitability of a cornea for transplantation is assessed by the corneal surgeon, who will consider the donor screening report, slit lamp and specular microscopic results, and serology reports. Following processing and evaluation of corneas and serological testing, transplantable corneas are transported to hospitals individually sealed and packaged, maintaining the cold chain at four degrees Celcius. The vial containing the cornea must be labelled properly with the eye bank name, tissue number, name of the preservative medium, medium lot number, expiry date of the medium, and date and time of the donor's death. The surgeon must also be provided with the donor screening, tissue evaluation, and serology reports. It is important that the eye bank follows a fair and equitable system of tissue distribution.

## Standards

Eye banks should develop and adhere to acceptable standards. This reduces the risk that grafts will fail or that infection will be transmitted. It may help to refer to the technical guidelines and acceptable minimum medical standards of the European Eye Banking Association (see Useful Resources, page 38).

## Finding donors

Even with an effective eye bank, finding enough people willing to donate their corneas can be difficult.

Public awareness programmes play an important role. They must emphasise that corneas can be donated by anyone, whatever their age, religion, or gender, and that neither enucleation nor in situ corneal excision causes disfigurement of the face or any delays in funeral arrangements. Family pledging is also becoming more important as family consent is usually needed before eyes or corneas can be removed.

Some of these problems may be circumvented by favourable legislation for eye donation, such as a ‘required request' law. This law requires hospital authorities to identify potential cornea donors and obtain consent from bereaved family members. Another law employed in some countries, such as the United States and Ethiopia, is a ‘presumed consent' law. Under this law, every person who dies while in hospital is presumed to be an eye donor unless this is actively rejected by their next of kin.

**Figure:**
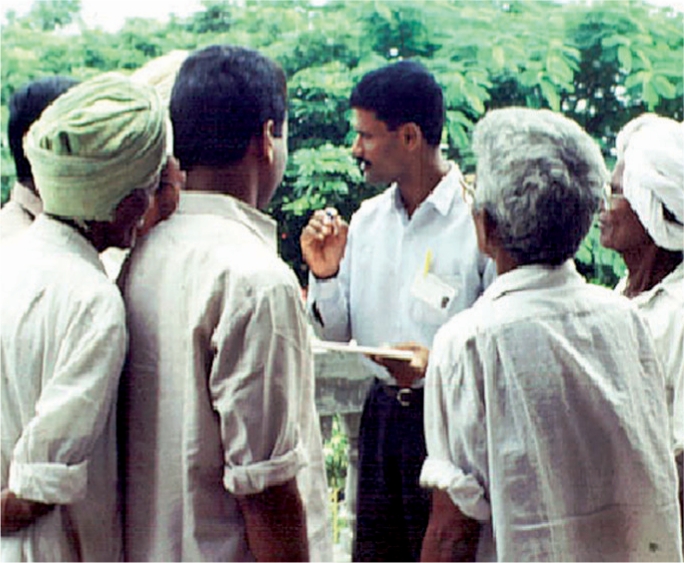
An eye donation counsellor speaks to family members of a deceased person. INDIA

Ramayamma International Eye Bank, LVP

Hospital cornea retrieval programmes can meet some of the immediate need. In these programmes, trained eye donation counsellors approach family members of the deceased and motivate them to consider eye donation. Training these counsellors in the art of grief counselling assists them in approaching family members at an appropriate time, sharing their grief, and preparing them to take the positive step of giving permission for eye donation on behalf of their loved one.
